# Preliminary analysis of proteome alterations in non-aneurysmal, internal mammary artery tissue from patients with abdominal aortic aneurysms

**DOI:** 10.1371/journal.pone.0192957

**Published:** 2018-02-22

**Authors:** Christina Lund Kidholm, Hans Christian Beck, Julie Bukh Madsen, Nikolai Bjødstrup Palstrøm, Jes Sanddal Lindholt, Lars Melholt Rasmussen

**Affiliations:** 1 Centre of Individualized Medicine in Arterial Disease (CIMA), Odense University Hospital, Odense, Denmark; 2 Department of Clinical Biochemistry and Pharmacology, Odense University Hospital, Odense, Denmark; 3 Department of Vascular Surgery, Odense University Hospital, Odense, Denmark; Stellenbosch University Faculty of Medicine and Health Sciences, SOUTH AFRICA

## Abstract

**Objective:**

The pathogenesis of abdominal aortic aneurysms (AAA) involves a disturbed balance of breakdown and buildup of arterial proteins. We envision that individuals with AAA carry generalized arterial protein alterations either because of effects of genetically or environmental AAA risk factors or because of compensatory changes due to signaling molecules released from the affected aneurysmal tissue.

**Approach:**

Protein extraction and quantitative proteome analysis by LC-MS/MS (liquid chromatography-mass spectrometry) was done on individual samples from the internal mammary artery from 11 individuals with AAA and 33 sex- and age-matched controls without AAA. Samples were selected from a biobank of leftover internal mammary arterial tissue gathered at coronary by-pass operations.

**Results:**

We identified and quantitated 877 proteins, of which 44 were differentially expressed between the two groups (nominal p-values without correction for multiple testing). Some proteins related to the extracellular matrix displayed altered concentrations in the AAA group, particularly among elastin-related molecules [elastin, microfibrillar-associated protein 4 (MFAP4), lysyl oxidase]. In addition, several histones e.g. (e.g. HIST1H1E, HIST1H2BB) and other vascular cell proteins (e.g. versican, type VI collagen) were altered.

**Conclusions:**

Our results support the notion that generalized alterations occur in the arterial tree in patients with AAA. Elastin-related proteins and histones seem to be part of such changes, however these preliminary results require replication in an independent set of specimens and validation by functional studies.

## Introduction

Abdominal aortic aneurysms (AAA) are often asymptomatic until they rupture with a mortality of around 80–90% [1]. AAA is estimated to be the tenth most common cause of mortality in the Western world [2]. Ultrasound is the gold standard for identifying AAA’s and in several countries introduced as a screening program for high risk individuals [3, 4]. The pathogenesis behind the disease is largely unknown, and no medical treatment is available and elective surgery is associated with 1.5–3.5% risk of death.

The pathogenesis behind AAA is multifactorial with interplay between genetic predisposition and environmental risk factors, such as cigarette smoking, male sex, aging, hypertension and inflammation [2, 5]. Although intimal atherosclerotic lesions may be a prerequisite for initiation of aneurysm formation, the presence of aortic atherosclerotic plaques is only weakly associated with aortic dilatation, suggesting that atherosclerosis is not the most important event in AAA development [6]. The putative genetic contribution to the disease is still not clear, but in a large population-based twin study, Wahlgren et al. found that the heritability of AAA is over 70% and further that the relative risk of developing AAA for first-degree relatives to persons diagnosed with AAA was doubled, compared to persons with no family history [2, 7]. Several genes have been identified as associated to AAA development, but around 80% of the inter-individual variation in AAA progression needs still to be explained [8]. Also epigenetic factors, such as histone alterations and DNA-methylations have recently been suggested as contributors to AAA development [8–11].

The pathogenesis behind the disease is incompletely understood, but it is known that degradation of the elastic media with remodeling of the aortic extracellular matrix proteins (ECM) occur, which ultimately leads to dilatation of the aortic wall due to largely unknown mechanisms [12, 13]. Matrix metalloproteinases (MMPs) are a family of proteases involved in degeneration and remodeling of ECM, and there have been suggestions that a series of polymorphisms in these proteins are related to the development of AAA [14]. In several studies it is found that especially MMP2 and MMP9 seem to have an important role in degrading elastin and collagen [15–18]. A recent meta-analysis identified some AAA-risk loci using genome-wide association studies (GWAS), and found a possible connection to MMP9 [19]. However, besides the well-characterized elastolysis and collagenolysis little is known about putative changes in other ECM proteins. A few studies have been performed on protein composition of the arterial wall in AAA. These proteome studies have investigated the protein composition in the heterogeneous tissue from aneurysms from patients operated for an AAA [20–25] and suggested some ECM proteins and other vascular molecules to be differentially expressed. Proteome studies have also been done on plasma samples from patients with AAA [26] revealing potential biomarkers, however no studies have focused on putative generalized proteome changes throughout the arterial tree among patients with AAA.

In the present study, we hypothesized that the risk of developing AAA is partly based on the general constitution of proteins in the arterial wall. We think that the arterial protein composition is influenced by genetic and environmental factors and that alterations in both ECM and vascular cell proteins may favor aneurysm formation when other local factors also are at play. We believe that such generalized arterial protein alterations may be revealed by proteome analysis of non-affected arterial tissue from patients with AAA. The internal mammary artery has proven to be a suitable model artery for investigations of generalized non-atherosclerotic arterial changes, for example in relation to diabetes [27], arterial stiffness [28] and smoking [29]. This artery has also been shown to reflect acute generalized effects of statin treatments [30]. Moreover, the degree of ECM accumulation and fibrosis in the internal mammary artery, as estimated by histology is closely related to the degree of fibrosis in other vessels, as for example in the coronary and carotid artery [31].

## Material and methods

### Patients and methods

#### Patients and arterial material

Since 2008 the spare material from the internal mammary artery from elective coronary bypass operations has been collected at the Department of Thoracic, Heart and Vascular Surgery, Odense University Hospital and has been stored in the Odense Artery Biobank. After the tissue has been collected from the operation room, the artery was dissected free from the surrounding adventitia, fat and muscle, by a trained technician unaware of the aneurysm status of the patient. The arteries were snap frozen and stored at -80°C until further analysis.

#### Patient selection

Patients registered in Odense Artery Biobank were coupled to The Danish Vascular Registry (Karbase) in order to identity patients with a recognized AAA. Three matching controls per AAA case were identified in the biobank, matching for age at surgery, sex, smoking and diabetes status. For the current study, arterial tissue from 11 patients with an AAA was available from our tissue collection, together with arteries from a group of 33 matched controls. The patients were defined as having type 2 diabetes (T2DM), if this diagnosis was registered in the patients file, or if at least one HbA1c measurement was above 6.5% (48 mmol/mol) or if the patients received anti-diabetic drugs and if they were found negative for GAD-antibodies in a blood sample taken the day before the operation. Current smoker is defined as the patient is actively smoking or has stopped smoking less than 6 months ago. Former smoker is defined as the cases where a patient has stopped smoking for at least 6 months. The patients were defined as having an AAA if they had an anterior-posterior aortic diameter > 30 mm, measured by ultrasound. Information about clinical parameters and medications were obtained from patient files. All participants gave written informed consent, and the study was approved by The Regional Committees on Health Research Ethics for Southern Denmark (S-20140202).

### Material preparation and analysis

#### Protein extraction

Proteins were extracted by transferring artery tissue (app. 3-30mg) to an Eppendorf tube containing 50 μl extraction buffer. The Eppendorf tube was incubated for 2x15 min in ultrasonic bath, followed by 20 min at 99°C and 120 min at 80°C in a heating block with 600rmp agitation. Extracted proteins were alkylated by adding iodoacetamide (IAA) to a final concentration of 0.2 M, followed by incubating for 30min in the dark at room temperature. The extracted proteins were precipitated by acetone precipitation, and to the resulting protein pellets were added 8 M urea, and 0.2 M TEAB. The protein digesting was performed by adding trypsin (Novo) by overnight incubation at 37°C.

#### Proteome analysis

Based on a nanodrop protein measurement of the tryptic digest, 10 μg peptides from each patient were labeled with the Tandem Mass Tag (TMT) 6 –plex reagent according to the manufacturer’s description (Thermo Science, Rockford, IL, USA.)

The samples were labeled as follows: Label 126: pool of all 44 samples, 127: case AAA, 128: control 1, 129: control 2, 130: control 3. The labeled peptides were mixed in equal amounts into a total of 11 6-plex samples, and each sample was fractioned using Hydrophilic Interaction Liquid Chromatography (HILIC). LC-MS/MS analyses were performed on these mixed samples with 4 individual samples (and one pool) per run.

#### Reversed phase desalting of peptides

To exchange the buffer and remove salts before the hydrophilic interaction liquid chromatography (HILIC) fractionation, the acidified samples were desalted using a reversed phase micro column packed in a Gel Loader tip using a 3M Empore C18 disc (3M Bioanalytical Technologies, St. Paul, MN) and a 50/50 mixture of Poros R2 and Poros Oligo R3 reversed-phase material (PerSeptive Biosystems, Framingham, MA) essentially as described in Gobom et al. [32].

#### Off-line HILIC fractionation

The HILIC fractionation was performed essentially as described in Beck et al. [33]. In brief, the peptides were dissolved in HILIC buffer B [90% (v/v) acetonitrile, 0.1% (v/v) TFA] fractionated into 10 fractions using a Dionex UltiMate 3000 nano HPLC using a 42 min linear gradient and a TSKgel amide-80 HILIC column.

#### On-line reversed phase LC separation and mass spectrometric analyses

The mass spectrometric analyses of the HILIC fractions and the validation study were performed on a Q-Exactive (Thermo Scientific) instrument coupled to a Dionex UltiMate 3000 nano HPLC, essentially as described in Beck 2011 [34]. The LC separation of the peptides was carried out using a custom made fused capillary pre-column (2 cm length, 360 μm OD, 75 μm ID) with a flow of 4 μl per min. for 7 min. Trapped peptides were separated on a custom made fused capillary column (15 cm length, 360 μm outer diameter, 75 μm inner diameter) packed with ReproSil Pur C18 3-μm resin (Dr. Maish, GmbH) with a flow of 250 nl per min. using a linear gradient from 95% solution A [0.1% formic acid (FA)] to 35% B [100% Acetonitrile(ACN) in 0.1% FA] over 30 or 86 for the discovery study, and 59 min for the protein verification study, followed by 10 min. at 90% B and 5 min. at 98% A. Mass spectra were acquired in positive ion mode applying automatic data-dependent switch between an Orbitrap survey Mass Spectrometry (MS) scan in the mass range of 400 to 1500 m/z followed by High Energy Collisional Dissociation (HCD) fragmentation and Orbitrap detection of the 15 most intense ions observed in the MS scan.

#### Data processing and quantification

All Q-Exactive raw data files were processed and quantified using Proteome Discoverer version 1.4.0.288 (Thermo Scientific). The Sequest search engine and Mascot search Engine (v. 2.2.3) both integrated with Proteome Discoverer were used to search the data with the following criteria—protein database: Uniprot/Swissprot (downloaded 7th November 2012, 452768 entries) and restricted to humans. Fixed search parameters included trypsin, one missed cleavage allowed, and carbamidomethylation at cysteine, while methionine oxidation and deamidation were set as dynamic. TMT 6-plex as fixed modification (K and N-terminal) was also applied as fixed modification for the discovery experiment. Precursor mass tolerance was set to 10 ppm and fragment mass tolerance was set to 0.1 Da. Peptide data were extracted using Mascot significance threshold 0.05 and minimum peptide length 6. For the discovery experiment a minimum of 2 peptides were used for protein identification and for protein quantitation, and for the validation experiments the mean of peak areas of the three most abundant precursor ions using the Precursor Ions Area Detector node in Proteome Discoverer. FDR was calculated using a decoy database search and only high confidence peptide identifications (False discovery rate < 1%) were included. For the discovery experiment protein quantification of the individual samples was done relative to the pool of arterial material from all 44 individuals.

#### Statistical analysis

Values are displayed as mean +/- standard deviation (SD), or as a number when appropriate. Student’s t-test was used to determine statistical significance between groups, and a p-value < 0.05 was considered statistically significant, however p-values were not corrected for multiple testing. For comparison of categorical variables, Fisher’s exact test or the Chi Square Test analysis was used.

## Results

Baseline patient characteristics for the two groups are presented in Table 1 reflecting a typical AAA patient population; 90% of the patients were male, with a current smoking percentage of almost 40%, and a mean age around 72 years. There were no significant differences in clinical parameters between the two groups regarding age at surgery, diabetes, Body Mass Index (BMI), sex, smoking status, medical treatment or diastolic blood pressure, but patients with an AAA had significantly lower systolic blood pressure (p<0.05).

**Table 1:** Clinical characteristics of patients with abdominal aortic aneurysm (AAA) and patients without abdominal aortic aneurysm (Non-AAA).

	AAA (N = 11)	Non-AAA (N = 33)	*p*-value
Age at surgery (years)	71.7 ± 7.9	71.6 ± 8.0	0.97
Male/female	10/1	30/3	1.0
BMI (kg/m^2^)	27.2 ± 3.5	27.4 ± 3.2	0.86
DM type 2 (yes/no)	2/9	6/27	1.0
Smokers current/former	4/7	13/19	1.0
Cholesterol (mmol/L)	4.0 ± 0.9	4.3 ± 1.0	0.38
Hypertension (yes/no/NA)	7/0/4	17/4/12	0.89
Systolic BP (mmHg)	128.7 ± 25.4	146 ± 22	0,043
Diastolic BP (mmHg)	69.4 ± 8.6	75 ± 12	0.16

The data is presented as mean ±SD or numerical when appropriate. NS = Non-Significant. BMI = Body Mass Index, DM = Diabetes Mellitus, BP = Blood Pressure, NA = Information not available

Using liquid chromatography tandem mass spectrometry-based (LC-MS/MS) proteomic analysis to investigate the protein composition of the arterial wall, we were able to identify and quantitate 877 proteins, as presented in S1 Table. Of these proteins, 44 showed significantly different levels, when arteries from patients with AAA were compared to tissue from individuals without AAA [p<0.05, t-test (not corrected for multiple testing)].

**Fig 1:**
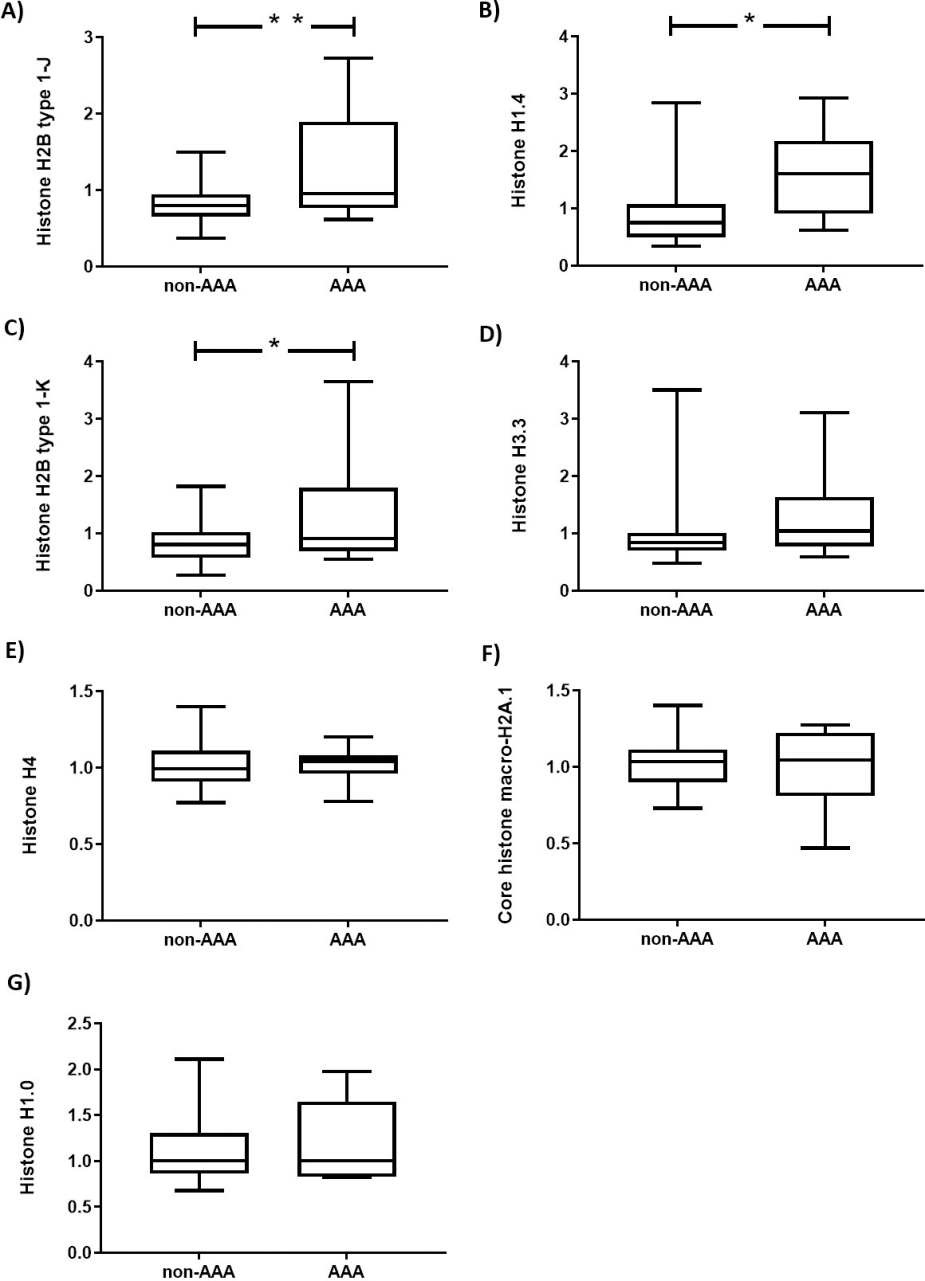
The relative amount of histones in patients with and without AAA. Histone H2B type 1-J(A), histone H1.4 (B), histone H2B type 1-K (C), histone H3.3 (D), histone H4 (E), core histone macro-H2A.1 (F) and histone H1.0 (G). Data are presented as median, interquartile and range. ** indicates p<0.001, * indicates p<0.01 (without correction for multiple testing).

Of the differentially expressed proteins, 32 had higher levels (Table 2A), and 12 proteins had lower levels among patients with AAA (Table 2B). Four of the proteins with most significant higher levels belong to the histone family, as can be seen in Table 2A. In the complete list of identified proteins (S1 Table), it can be seen that seven are histones and of these, three were regulated and four were not, as shown in Fig 1. Using a Fisher’s exact test, it can be calculated that the distribution of proteins with altered versus non-altered protein amounts in the histone group (3 versus 4) was highly significant different from what could be expected among non-histone proteins (41 regulated and 830 non-altered non-histone proteins, p = 0.0036).

**Table 2:** Comparison of protein levels in the internal mammary artery between AAA-cases and controls.

Regulated proteins		Non-AAA		AAA			
Uniprot accession number	Protein name	Mean	SD	Mean	SD	Fold change	P-value*
**A. Higher levels in AAA patients**							
P00325	Alcohol dehydrogenase 1B	0.85	0.258	1.18	0.286	1.38	0.001
P31946	14-3-3 protein beta/alpha	0.79	0.339	1.67	1.090	2.12	0.001
P06899	Histone H2B type 1-J	0.81	0.239	1.34	0.759	1.65	0.001
P10412	Histone H1.4	0.90	0.553	1.62	0.728	1.80	0.002
O60814	Histone H2B type 1-K	0.83	0.329	1.35	0.955	1.64	0.006
Q9Y5Z4	Heme-binding protein 2	0.82	0.303	1.34	0.494	1.63	0.006
F5H265	Ubiquitin (Fragment)	0.93	0.268	1.44	0.585	1.54	0.008
P39656	Dolichyl-diphosphooligosaccharide—protein glycosyltransferase 48 kDa subunit	1.00	0.170	1.21	0.242	1.20	0.008
P21926	CD9 antigen	0.90	0.260	1.39	1.000	1.54	0.010
Q16555	Dihydropyrimidinase-related protein 2	0.96	0.193	1.24	0.523	1.28	0.011
P06396	Gelsolin	0.92	0.119	1.04	0.170	1.13	0.012
Q05707	Collagen alpha-1(XIV) chain	0.93	0.152	1.09	0.252	1.17	0.013
O75531	Barrier-to-autointegration factor	1.07	0.223	1.44	0.229	1.34	0.014
P69905	Hemoglobin subunit alpha	0.90	0.302	1.23	0.572	1.36	0.014
P13611-2	Isoform V1 of Versican core protein	0.91	0.328	1.40	0.667	1.54	0.014
Q15847	Adipose most abundant gene transcript 2 protein	0.75	0.339	1.35	0.900	1.79	0.015
P50238	Cysteine-rich protein 1	0.79	0.505	1.44	1.394	1.82	0.020
E7ENM0	Elastin	0.80	0.426	1.26	0.876	1.56	0.020
O60237-4	Isoform 4 of Protein phosphatase 1 regulatory subunit 12B	0.77	0.214	1.74	0.826	2.27	0.022
P55083	Microfibril-associated glycoprotein 4	0.96	0.283	1.21	0.412	1.26	0.025
P07602	Proactivator polypeptide	0.94	0.293	1.31	0.822	1.38	0.028
P31949	Protein S100-A11	0.98	0.227	1.19	0.367	1.21	0.031
P62906	60S ribosomal protein L10a	0.95	0.094	1.20	0.166	1.26	0.031
P53708	Integrin alpha-8	0.97	0.205	1.14	0.216	1.18	0.032
Q08397	Lysyl oxidase homolog 1	0.96	0.240	1.18	0.382	1.23	0.036
P06753	Tropomyosin alpha-3 chain	1.03	0.270	1.52	0.836	1.47	0.037
Q14195-2	Isoform LCRMP-4 of Dihydropyrimidinase-related protein 3	0.95	0.316	1.45	1.082	1.53	0.037
P39019	40S ribosomal protein S19	0.99	0.255	1.23	0.161	1.25	0.039
Q99536	Synaptic vesicle membrane protein VAT-1 homolog	0.92	0.170	1.10	0.281	1.19	0.042
Q6UXI9	Nephronectin	0.96	0.315	1.26	0.531	1.32	0.044
P37802	Transgelin-2	1.00	0.208	1.17	0.359	1.17	0.048
P55072	Transitional endoplasmic reticulum ATPase	0.99	0.149	1.10	0.099	1.10	0.049
**B. Lower levels in AAA patients**							
P31943	Heterogeneous nuclear ribonucleoprotein H	1.13	0.169	0.93	0.167	0.83	0.005
P06576	ATP synthase subunit beta, mitochondrial	1.08	0.144	0.94	0.170	0.87	0.009
P00505	Aspartate aminotransferase, mitochondrial	1.17	0,178	1.01	0.157	0.87	0.020
Q13561	Dynactin subunit 2	1.12	0.200	0.95	0.118	0.84	0.027
P12109	Collagen alpha-1(VI) chain	1.03	0.175	0.91	0.114	0.88	0.028
P13489	Ribonuclease inhibitor	1.09	0.227	0.92	0.165	0.84	0.036
P34932	Heat shock 70 kDa protein 4	1.04	0.181	0.86	0.067	0.82	0.037
Q9BVC6	Transmembrane protein 109	1.03	0.173	0.90	0.249	0.87	0.038
P35268	60S ribosomal protein L22	1.10	0.163	0.69	0.303	0.63	0.042
Q6IBS0	Twinfilin-2	1.12	0.228	0.78	0.238	0.69	0.048
P14543	Nidogen-1	1.09	0.214	0.94	0.208	0.87	0.049
P55786	Puromycin-sensitive aminopeptidase	1.07	0.184	0.94	0.134	0.87	0.049

*p-values are as calculated by simple t-tests and not corrected for multiple testing

**Fig 2:**
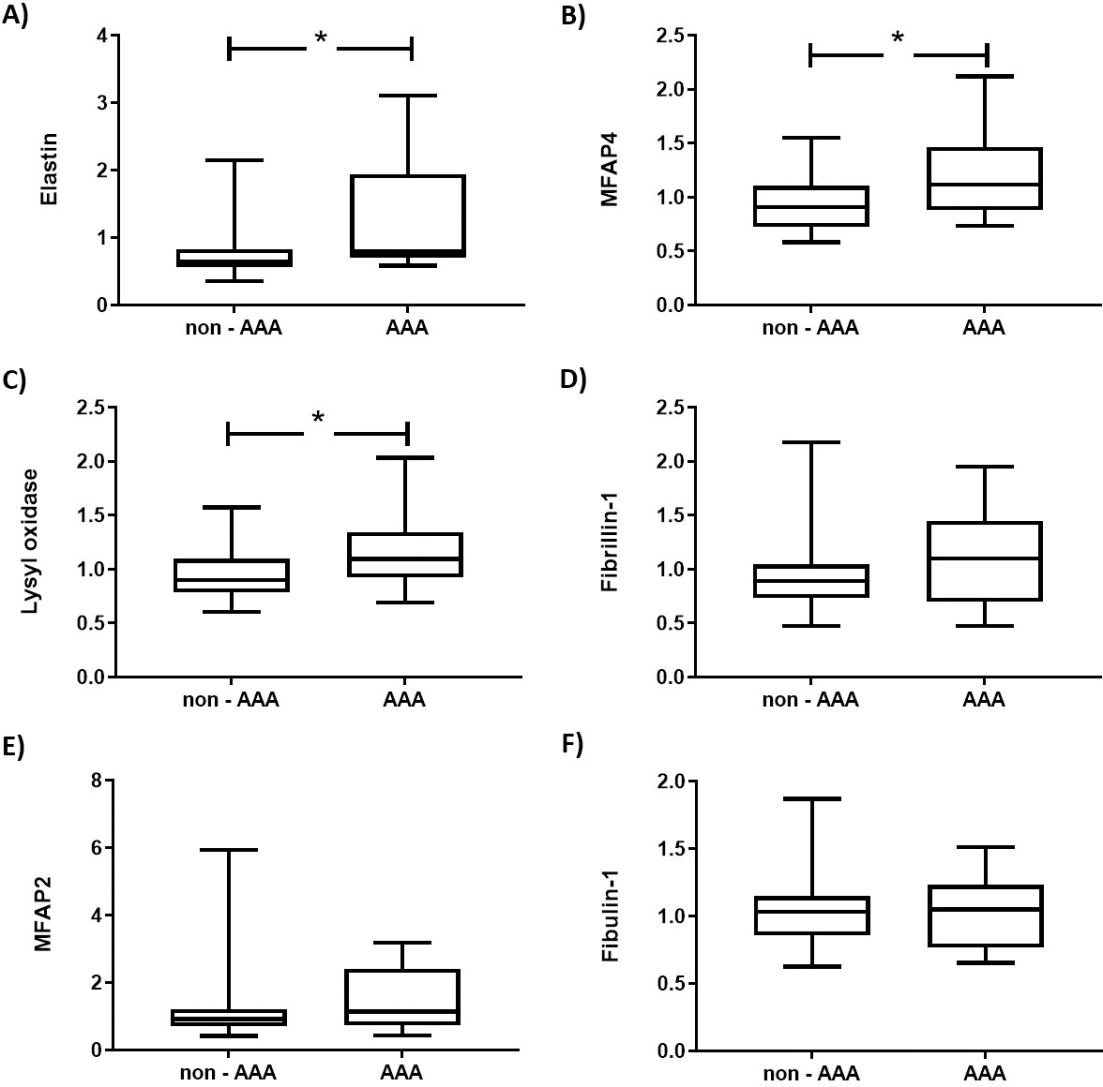
The relative amounts of elastin-related arterial proteins. Elastin (A), MFAP4 (B), lysyl oxidase (C), fibrillin-1 (D), MFAP2 (E) and fibulin-1(F) in patients without abdominal aortic aneurysm (non-AAA) or with abdominal aortic aneurysm (AAA). Data are presented as median, interquartile and range * indicates p<0.05 (without correction for multiple testing).

Three of the proteins with significantly elevated levels in AAA belonged to the group of elastin-related proteins, as can be seen in Table 2A. In the list of identified proteins (S1 Table), six elastin-related proteins were identified and of these, three had increased arterial levels and three not, as shown in Fig 2. Using a Fisher’s exact test, we calculated that the distribution of proteins with increased levels versus non-altered proteins in the elastin-related proteins group (3 versus 3) was highly significantly different from what could be expected (41 with significantly altered levels of 829 non-regulated non-elastin-related proteins, p = 0.0021).

In addition to histone-related and elastin-related proteins, it was also worth noticing that among proteins with the highest degree of up-regulation (fold change) are 14-3-3 protein beta/alpha, ubiquitin and isoform 4 of protein phosphatase 1 regulatory subunit 12B. Also noteworthy, some extracellular matrix proteins were regulated, i.e. collagen type XIV, versican and nephronectin (higher arterial amounts in AAA) as well as collagen type VI and nidogen-1 (lower arterial amounts).

To verify some of the protein results, we performed an additional LC-MS/MS run on all samples using label-free quantification on remaining protein digests. We verified the presence of other peptides for proteins in the elastin-related-protein group. Although quantitation by this method in our samples is considerably more imprecise than the above used method with TMT-labelling, we were able to find results displaying comparable results, although some do not reach significance (elastin: 63+/-4.2 (AAA) vs 35+/-2.7 (non-AAA) (p = 0.01); MFAP4: 51+/-3 (AAA) vs 37+/-2.8 (non-AAA) (p = 0.15); Lysyl oxidase: 31+/-9 (AAA) vs 29+/-2.2 (non-AAA) (p = 0.88); fibrillin-1: 44+/-3 (AAA) vs 32+/-2.4 (non-AAA) (p = 0.0.15); MFAP2: 20+/-9.3 (AAA) vs 13+/-9.6 (non-AAA) (p = 0.07); fibulin-1: 13+/-6 (AAA) vs 16+/-7.9 (non-AAA) (p = 0.01) (results are presented in arbitrary units as mean+/-SD).

## Discussion

In this study, we hypothesized that patients with AAA display global arterial protein changes and that information concerning such generalized changes could be extracted by proteome investigations of non-affected arterial tissue from patients with AAA. Knowledge about generalized arterial changes may point toward important mechanisms behind the development of aneurysms. The number of investigations of the molecular pathology of human arterial tissue are sparse, because of the difficulty of accessing relevant material: However, due to a systematic gathering of the internal mammary artery from more than 1000 coronary by-pass operations, it has been possible to obtain a suitable number of samples from individuals with AAA among the by-pass operated patients. Our earlier investigations have shown that the internal mammary artery harvested from by-pass operations is homogenous, free of atherosclerosis and useful for proteome analysis, which is the background for our previous results showing distinct arterial protein changes in relation to diabetes, smoking and stiff arteries [28, 29, 35]. In this study we were able to quantitate several hundred arterial proteins using iTRAQ-labelling and we found alterations among several molecules among patients with AAA. Moreover, a selected subset of these proteins could be confirmed by another label-free LC-MS/MS methods.

We expected only subtle generalized arterial alterations in the vascular tree among patients with AAA, which is in line with the fact that we only observed a limited number of arterial proteins with altered concentrations among patients with AAA. None of these results were significant after multiple testing correction in our study, and although our samples are very homogenous and the variation of protein expression is low, a much larger study would be needed to obtain corrected significance with the observed rather subtle changes. The obtained results concerning single proteins were therefore preliminary and need confirmation in other cohorts. Nevertheless, among the differentially expressed molecules, a statistically significant number of proteins displayed alterations in two specific gene families. Firstly, among identified histones, we observed up-regulation in 4 out of 8 identified molecules in this family. Histones are part of the epigenetic machinery and it is therefore interesting that epigenetic factors like histone alterations and DNA-methylations were recently suggested as contributors to AAA development [8–10]. Moreover, earlier studies have reported a significant difference in the amount of the enzyme acetyltransferase, responsible for the acetylation of the histones in AAA [10]. The exact importance of these findings is not yet known, however our findings are compatible with the idea that epigenetic changes in vascular cells may be involved in the development of aneurysms.

Secondly, we observed that 3 out of 6 of the proteome-identified elastin related molecules [elastin itself, lysyl oxidase (LOX) and microfibril-associated protein 4 (MFAP4)] are up-regulated in the arterial tissue from patients with AAA. Structural, biochemical and also genetic studies have shown that elastin degradation plays an important role in aneurysm formation [36–38]^,^ and aneurysms can be induced by experimental injection of porcine pancreatic elastase, although this may relate to other components than the elastase itself [39]. Nevertheless, it is of interest that increased synthesis of matrix molecules, including elastin accompanies aortic aneurysms, probably as a compensatory mechanism [40, 41]. This notion fits well with experimental observations showing that adipose stem cells promote vascular smooth muscle cells elastin production during aneurysm formation [42], and with data to the effect that gene therapy with adenovirus mediated tropoelastin expression may be beneficial in AAA [43]. Our proteome data showed that elastin is upregulated, which were supported by the fact that also elastin-related proteins were upregulated. MFAP4 is an elastin binding matrix molecule, abundantly distributed in arteries, where it plays an important role in elastin-assembly, but also in the migration of vascular smooth muscle cells [44]. Moreover, LOX is an enzyme, catalyzing cross-linking of collagens and elastin, which is essential for stabilization of collagen fibrils and for the integrity and elasticity of mature elastin. Animal studies in which lysyl oxidase was inhibited resulted in lathyrism, characterized by poor bone strength, weak ligaments, and increased occurrence of aortic aneurysms [45]. Our observations of increased amounts of elastin and elastin-related molecules in non-affected arterial tissue among patients with AAA support and expand the elastin compensation theory, and suggest that the responsible factor(s) may be paracrine or endocrine. This aspect could potentially be of use in the development of future medicinal targets against aneurysm formation, but needs of course more studies.

A few of the identified differentially regulated proteins have been considered in relation to AAAs. Versican, which we found increased has previously been found in decreased amounts in affected tissue from a few persons with AAA [46]. Likewise, collagen type VI, which we found in decreased amounts, has been shown to correlate with the growth rate of AAA in aneurysmatic tissue [21]. The significance of these and other regulated genes remains to be investigated.

Our study suffers from several limitations. Although we have access to a large number of arteries, only 11 of the by-pass operated patients had an AAA, and to improve power of the study, we therefore selected a threefold larger non-AAA group. Thus, the relatively small patient number and subtle protein changes did not allow for corrections for multiple testing in the statistical analysis and the presented results therefore need confirmation in other cohorts. Secondly, all the tissue samples in our study come from patients undergoing coronary by-pass surgery, which indicates a certain degree of atherosclerotic disease throughout the arterial tree among all patients. Nevertheless, the mammary artery itself does not contain atherosclerotic plaques [35] and although our results could be influenced by variations related to the general degree of atherosclerotic disease, we think that it is highly likely that the observed differences reflect the coexistence of aneurysmatic disease, since all patients have coronary disease.

The presented results indicate that subtle generalized protein changes may be present in non-affected arteries in patients with AAA. Alterations occurred particularly in histones and elastin-related proteins, but other vascular proteins displayed different concentrations as well. Further studies are needed to determine if the observed preliminary changes are causative, compensatory or only associatively involved in the development of AAA.

## Supporting information

S1 Table Quantitative proteome data for all identified proteins in patients without (Non–AAA) and with abdominal aortic aneurysm (AAA) Proteins quantified using TMT isobaric mass tags. Protein extracts from arteries from 33 Non-AAA controls and 11 AAA case individuals were processed as described in the methods sections, randomly labeled with six-plex TMT isobaric tags (mass tags 127–130) and analyzed by nano-LC MS/MS. The individual samples were compared with a pool of all 44 samples (TMT tag 126). The resulting ratios for each protein were used for student’s t-test calculation. Columns indicate Uniprot accession number, Protein function, Clinical parameters, the percentage of matching amino acids from identified peptides, the sum of unique peptides used for identification, the theoretical protein molecular weight and the calculated isoelectric point. Blanks indicate that the specific protein was not identified in this particular experiment. Samples from from patients with AAA are indicated with AAA in row 2, whereas patients without AAA are indicated with C.
(XLSX)
